# Prostatic Neoplasia in the Intact and Castrated Dog: How Dangerous is Castration?

**DOI:** 10.3390/ani10010085

**Published:** 2020-01-05

**Authors:** Magdalena Schrank, Stefano Romagnoli

**Affiliations:** Department of Animal Medicine, Production and Health (MAPS), University of Padua, 35122 Padova PD, Italy; stefano.romagnoli@unipd.it

**Keywords:** dog, castration, gonadectomy, prostatic neoplasms, prostate cancer

## Abstract

**Simple Summary:**

Castration of dogs is a routinely performed surgery to limit unwanted reproduction and prevent pathologies of the genital tract. Over the last two decades, the number of reports on possible long-term health risks has increased. Pet-owners have easier access to scientific publications and are concerned about reports on increased risks of castrated dogs for neoplastic diseases. Divulgation of results without consideration of study design and inclusion criteria for the studied populations may result in premature conclusions impacting many stakeholders. Our aim is to provide a detailed description of prostatic cancer in the dog and the possible side effects of castration. Age at diagnosis ranges from 8.5 to 11.2 years in both intact and castrated dogs. A cytological or histological exam is needed to confirm a suspect. Most dogs already present metastasis at the time of diagnosis which makes prognosis generally poor, also if lung metastasis reportedly has no negative impact on the survival time. Castrated dogs with prostate cancer have been reported to live longer than intact ones. We conclude that until today, we knew too little to exclude routine castration of adult male dogs under six years of age from the veterinary practice due to concerns of causing prostatic neoplasia.

**Abstract:**

Elective gonadectomy in the dog is a topic of interest for clinicians, pet-owners, and society. Although canine prostatic neoplasia (CPN) has a low incidence (0.35%), reports of an increased risk for castrated dogs attract attention and cause concern in pet-owners. Our aim is to provide professionals and non-professionals with a detailed description of this possible side effect of gonadectomy in the dog. The mean age at diagnosis of CPN ranges from 8.5 to 11.2 years. Medium to large size breeds are more frequently affected. Symptoms and findings of non-invasive examinations are not pathognomonic, therefore, cytological or histological examinations are needed for diagnosis. Overall, the incidence of metastasis reaches up to 80%, yet lung metastasis reportedly has no negative impact on median survival time (MST). It has been reported that castrated males have a significantly higher MST than intact males. Differences in inclusion criteria for studied populations make a comparison of studies difficult. Citation of odds ratios without consideration of the context of the reference may result in premature conclusions. We conclude that elective gonadectomy of adult male dogs under six years of age cannot be excluded from the veterinary practice due to concern of causing CPN until clear and strong evidence is available.

## 1. Introduction

Elective gonadectomy is most commonly used for the prevention of unwanted reproduction in cats and dogs. Especially in countries with a high number of stray animals, neuter-spay-programs have been initiated and owners have been advised to have a surgical castration performed on their pet. Although there are clear and well-documented advantages of gonadectomy in the dog, the reports of possible disadvantages increased over the last two decades and cause concern in a large group of pet-owners. One such possible reported disadvantage is the canine prostatic neoplasia (CPN). Canine prostatic neoplasia has been attracting the interest of researchers and clinicians due to its similarities with human prostatic neoplasia despite a much lower incidence in dogs (0.35%) compared to humans (30%) based on necropsy studies [1,2]. The role of neuter status has been the object of ongoing debate because of claims that gonadectomy may increase the incidence and/or hasten the progression of prostatic neoplasia in male dogs. Over the last two decades, several studies have shown a statistically significant increased risk for the diagnosis of various neoplastic conditions in castrated males and females compared to intact dogs [3,4,5,6,7,8]. These findings, among others, have initiated a discussion on the advantages and disadvantages of elective gonadectomy in the dog. However, a definitive explanation of how the lack of gonadal hormones may influence the development of neoplasia in reproductive or non-reproductive tissues has yet to be provided. Several reviews have been published over the years on the advantages and disadvantages of gonadectomy in dogs [9,10,11,12,13]. However, two factors have had an impact on the quality of these publications: editorial limitations of the publication’s length and the tendency to report only the odds ratio, without considering the study design and the conclusions drawn by the authors of the cited publication [10,12,14]. Therefore, the role of neuter status on the development of canine prostatic neoplasia has been considered as a risk factor not only for prostatic neoplasia but also for other neoplastic disorders in male dogs, rising concerns on whether or not elective gonadectomy in dogs should be regarded as a safe procedure. The aim of this paper is to provide an in-depth review of current knowledge on canine prostatic neoplasia and the role of the castrated male. 

## 2. Incidence, Prevalence, and Signalment

A summary of the studies on canine prostatic neoplasia (CPN) is provided in Table 1. Weaver et al. [1] reported an incidence of canine prostatic neoplasia of 0.35%, more recently Bryan et al. [15] reported a maximum annual incidence of 0.93%, although the latter combine prostatic tumors with urethral and bladder transitional cell carcinoma (TCC). The incidence of CPN is not reported in any other study on this topic. Furthermore, the total number of animals is not always featured, as well as the type of neoplasia and the castration-diagnosis-interval (CDI). Although the total number of animals within a study varies widely (26–72,300 dogs), the retrospectively calculated prevalence (Ntotal/N of CPN = calculated prevalence) in most papers is <1% of the total population studied (Table 1). In the cases of Aquilina et al. [16], Troisi et al. [17], and Donato et al. [18], the study design may be an explanation for the high calculated prevalence of CPN. The total number of animals reported by these three publications is the number of animals included in the study rather than the total number of animals seen at the hospital during a certain period, making these values hardly comparable to prevalences calculated on the total population of dogs examined or autopsied. Bryan et al. [15] do not provide any information on overall incidence, yet report an annual occurrence of CPN ranging from 0% to 0.93%, with a mean and median annual occurrence of 0.45% and 0.47%, respectively. An important aspect of the signalment of dogs affected by prostatic neoplasia is age. Just as benign prostate hyperplasia (BPH) in intact males and pyometra in intact females, also prostatic neoplasia is a disorder mainly diagnosed in the elderly dog, with an average age of 8.5 to 11.2 years at the time of diagnosis (Table 1). Canine carcinomas are typical of elderly age [19] and it has been hypothesized that physiological changes due to aging may have an impact on the initiation of CPN development [20]. Although the median or mean body weight is rarely mentioned, medium to large size breeds are more frequently affected by CPN than small or miniature breeds [1,21,22]. No breed predilection has ever been confirmed. However, an increased risk/odds ratio of CPN has been reported for Shetland Sheepdogs, Scottish Terriers, Bouvier des Flandres, Dobermann Pinscher, and dogs of mixed breed [6,15,23,24]. 

### Role of Neuter Status

Obradovich et al. [29] and Bell et al. [30] were the first to investigate the role of castration in the development of CPN. A causal relationship was not postulated and until the end of the century, castration was widely believed to be the most appropriate treatment for any prostatic condition. However, over the last 20 years, there has been increased attention to the health consequences of gonadectomy in small animals and also CPN has been regarded as being influenced by castration in many reviews [9,10,12,14,41,42]. The sample size, number of dogs with CPN and CDI varies considerably among retrospective studies (Table 1). Unfortunately, few authors provide detailed information on (a) whether or not dogs were castrated, (b) when castration was performed during life, (c) the size of the reference population, (d) how long before the diagnosis the dog was castrated in order to be included in the castrated group. Sadly, most studies fail to provide information on (b) and (d). With regard to (d), only two authors provide detailed and complete information: Teske et al. [6] considered dogs as castrated if they were gonadectomized at least 100 days prior to diagnosis, yet reports a CDI ranging from 1–10 years; Obradovich et al. [29] instead used three years as a cut-off value after which the authors considered dogs as castrated at the time of diagnosis. If dogs were castrated less than three years prior to diagnosis, they were considered as sexually intact at the onset of prostatic disease. The majority of authors include castrated dogs in the group of castrates in their statistical evaluation despite lack of information on how recently (prior to diagnosis) castration was performed [16,23,27,33,34]. Sorenmo et al. [34] presented one of the few publications in which the number of castrated animals was higher than the number of intact animals. They report a median age at castration of two years (ranging from two to 14 years); information was unavailable in five cases, as was a median or mean value of the CDI. The interval between castration and diagnosis of prostatic neoplasia is vital information to investigate a possible correlation between gonadectomy and the incidence of prostatic neoplasia. Furthermore, the criteria on when a dog is included in the group of castrates should always be clearly stated. Age at diagnosis did not differ between castrated and intact dogs in the publications of Teske et al. [6] and Bell et al. [30], yet Bryan et al. [15] report that castrated dogs with prostatic neoplasia were significantly older at the time of diagnosis. Perhaps the decrease of prostatic size after castration that subsequently delayed onset of prostatomegaly related symptoms may cause a delayed diagnosis. Cornell et al. [32] instead report no difference in age at diagnosis.

## 3. Clinical Signs

Clinical signs may be grouped into gastrointestinal (GI, straining to defecate up to complete constipation, tenesmus, and deformed feces), urinary (straining to urinate, hematuria, incontinence, polyuria/polydipsia (PU/PD) and dysuria), and locomotor (hindlimb weakness, lameness and pain) [1,24,30,32,38,39]. Affected dogs may also show generalized symptoms such as anorexia, emaciation, weight loss, and abdominal pain. Whereas GI- and urinary tract signs may be easily explained on the basis of prostatic topographic anatomy, skeletal problems, and general signs are often secondary to metastatic disease. Table 2 shows the percentages of dogs that have at least one of the above-mentioned clinical signs. The majority of studies providing information on clinical signs describe almost all locomotor but not all systemic signs as being linked to the presence of metastasis. Bell et al. [30] report that urinary tract signs without GI-signs were found in 40% of neutered dogs but only in 9.5% of intact males, whereas GI-signs without urinary signs were found exclusively in intact patients, accounting for 33%, although the differences were not statistically significant. Dogs affected by BPH have been reported to show hematuria and urethral discharge, yet only a few of them show stranguria or dysuria [24] Although a high percentage of urinary tract signs is reported in dogs affected by prostatic carcinoma, the same clinical signs may be found in dogs affected by other prostatic disorders and may, therefore, not be considered CPN specific [24,41]. However small, animal practitioners should always consider CPN as a differential diagnosis in male dogs with severe urinary or GI-signs.

## 4. Diagnosis

Following a thorough clinical history and clinical exam, the performance of digital palpation of the prostate per rectum may give indications on the presence of a prostatic disorder [1,23,27]. In healthy dogs, digital rectal palpation should not elicit pain [44]. A prostate gland affected by neoplasia may be felt as an irregular, immobile, asymmetrical mass that is often (but not necessarily) painful [21]. Historically, radiology has been used to assess prostatic size [1,23,27,28] which is normal when its diameter does not exceed 50% or 70% of the width of the pelvic inlet [45,46]. Prostatic enlargement, calcification of the gland as well as bone and lung-metastases may be found in a latero-lateral projection [47]. Ultrasound and radiographic examination in dogs affected by CPN may show an enlarged gland with focal to diffuse hyperechoic areas, foci of mineralization, loss of physiologic prostatic contour, and locoregional lymphadenopathy [30,48,49]. A cytologic or histologic examination is necessary to confirm a suspected diagnosis of CPN. Cytological samples may be collected either by transabdominal fine-needle aspiration biopsy (FNAB) under ultrasound-guidance [50], or by cell collection via a urinary catheter following a prostatic massage. The collection of specimens for histology may be performed during laparotomy either as a punch-biopsy or excisional biopsy. Reliability of FNAB has been reported to vary between 50% [51] and 80% [37,52]. A biopsy followed by a histological examination resulted in correct diagnosis in 66–80% of cases [37,49,52,53]. The reliability of transabdominal FNAB and cell collection via a urinary catheter has been evaluated with the results being compared to biopsy results. FNAB correctly identified 63% of histologically diagnosed neoplasia, whereas catheter aspiration diagnosed 55% of cases correctly [53]. Transabdominal FNAB is currently not recommended by some authors, due to the possibility of dissemination of TCC tumor cells along the needle pathway [52]. Although excisional biopsies are contraindicated in the presence of acute prostatitis and abscesses [54], the technique is still considered the most reliable to diagnose CPN. Histologically CPN may be divided into prostatic adenocarcinoma (PACA), prostatic carcinoma (PCA) and tumors of mixed morphology [27]. PACA has been described as being either Type A (adenocarcinoma) or Type B (undifferentiated adenocarcinoma). Type A is subdivided into intra-alveolar proliferative or small acinar and Type B into syncytial or discrete epithelial [27]. The intra-alveolar proliferative type is reported to be the most common histological subtype. Tumors of mixed morphology were described as more frequently associated with metastasis [38]. Table 3 illustrates the prevalence of the different types of prostatic neoplasia. 

A marker expressed by the basal cell layer of the human prostate, P63, was found also in cases of CPN and the positivity of CPN for p63 was associated with a significant shortening of survival time [56]. Canine prostatic neoplasia with p63-positivity is suspected to present a distinct entity rather than a subtype of canine prostatic carcinoma [56]. Due to its possible implications on metastatic behavior, we consider the histological evaluation of CPN of utmost importance and, therefore, the biopsy is preferable towards cytological evaluations. The availability of an early marker for CPN would have an important impact on treatment and, therefore, the outcome and prognosis of the disease. Unfortunately, no early diagnostic marker for CPN is currently available. Canine prostatic arginine esterase (CPSE) is marketed as a diagnostic marker for prostatic disorders, yet until today, its measurement does not permit a distinction of CPN and other prostatic pathologies [31,57,58].

### Role of Neuter Status

Different authors describe the castrated male as being more frequently affected by poorly differentiated prostatic neoplasia [30,32,48,55]. Although Bryan et al. [15] report an increased overall risk for CPN in castrated dogs, PACA was the subtype with the least risk.

## 5. Metastatic Behavior

Prostatic neoplasia in both men and dogs is often associated with bone metastasis, giving the tumor the reputation to metastasize fast and frequently to the skeletal system [20]. However, when considering the reports on metastasis in dogs affected by CPN, lungs and regional (iliac) lymph nodes are affected more frequently than bones [1,22,27,30,32,34,43] (see Table 4).

The overall incidence of metastasis in dogs with CPN ranges from 16% [18] to 80% [32]. Canine prostatic neoplasia of mixed morphology was associated with an increased metastatic frequency [32,38]. These authors hypothesize that also the age of the patient influences the biological behavior of CPN. Prostatic tumors in the dog which stained positively for CK7 were reported to have both a higher overall metastatic frequency (*p* = 0.04) and a higher frequency of bone metastasis (*p* = 0.03), compared to CK7-negative CPN [34]. Surprisingly, lung metastasis has been reported to have no negative impact on median survival time (MST) [22]. Further research is needed to reevaluate the importance of the presence or absence of metastatic disease on treatment-choices and prognosis. 

### Role of Neuter Status

Castrated dogs had a statistically significant increase in lung metastasis frequency [30], yet Cornell et al. [32] report no difference in metastatic frequency or prevalence of skeletal metastasis between intact and castrated dogs. In the study of Sorenmo et al. [34] dogs with CK7 positive prostatic neoplasia had been castrated at a younger age than dogs with CK7 negative prostatic neoplasia; one could speculate that castration at a younger age increases the risk of metastatic disease and bone metastasis in cases of CPN.

## 6. Treatment and Outcome

Despite the improvement of prognosis in men thanks to recent advances in the diagnosis and treatment of prostatic neoplasia, prognosis, and outcome of this condition in dogs remain poor [42]. Early studies report a survival time in affected dogs of only a few days [1,20,23,30]. Treatment options include surgical removal of the tumor by subtotal prostatectomy or complete removal of the gland by total prostatectomy [40,59]. Radio-or chemotherapy protocols are described also for dogs affected by prostatic neoplasia [49]. However, because of diffuse metastatic disease at the time of diagnosis, the above treatment protocols are rarely applied [30,32,48]. Recently, Ravicini et al. [22] treated 67 dogs with various degrees of prostatic neoplasia either with nonsteroidal anti-inflammatory drugs (NSAIDs) (46%), with chemotherapy (6%), or with a combination of both (48%) (Table 5). None of the dogs in this study received surgical treatment or radiotherapy, yet 33% of these dogs showed improvement in clinical signs. The median survival time (MST) was 82 days with a range of 9–752 days. None of the 18% of dogs showing prolonged survival (>7 months) had metastatic disease. Lung metastasis had no negative impact on prognosis and MST, yet MST was negatively influenced by the sexual status with intact males living for a significantly shorter time than neutered males. Considering that already Gupta et al. [60] and Tremblay et al. [61] proposed a potential association between the presence of cyclooxygenase (COX)-2 and prostate carcinogenesis, the results of Doré et al. [62] on the enzyme expression and estrogen in the canine prostate may be considered of great importance due to its potential impact on the treatment of prostatic neoplasia. Surgical treatment is possible although urinary incontinence is reported as an important post-operative risk, mainly following total prostatectomy [21]. Bennett et al. [40] described permanent incontinence in eight out of 23 dogs treated with total prostatectomy. Only two of these incontinent dogs were affected by a prostatic carcinoma, whereas the other six dogs were diagnosed with a transitional cell tumor. L’Eplattenier et al. [59] used a surgical laser to perform a subcapsular partial prostatectomy (Table 5). Due to the incomplete removal of neoplastic tissue, all dogs in this study received a post-operative treatment with Interleukin-2 (which, however, was considered palliative rather than curative); incontinence was absent in all cases and the median survival time was 103 days with a range of 5–239 days [59]. In this paper [59] none of the animals had metastatic disease at the time of treatment. Bennett et al. [40] treated 25 dogs with total prostatectomy (Table 5). Of these 25 dogs, 15 were diagnosed with a TCC, nine with adenocarcinoma and one dog with undifferentiated carcinoma. The overall MST was 231 days, ranging from 24 to 1255 days, with one- and two-year survival rates equal to 32% and 12%, respectively, without significant difference between dogs affected by TCC and dogs affected by adenocarcinoma. Only one dog in this study had confirmed metastatic disease at the time of diagnosis. In order to improve the prognostic accuracy of CPN, the Gleason [63] scoring system was tested on its viability in the canine species [43]. A scoring system of 1–5 is given to the primary (most prevalent) and the secondary (second most prevalent) pattern found in a histological specimen. Tissue with grades 1–3 resemble normal prostatic tissue, while grades 4 and 5 refer to tissues with abnormal glandular architecture. Similar to men, dogs with metastatic disease had a Gleason score of 10, and therefore, a worse prognosis [43]. The Gleason scoring system may be used also in the canine species [43].

### Role of Neuter Status

Ravicini et al. [22] report that intact males with prostatic neoplasia had a significantly shorter median survival time than castrated males with prostatic neoplasia. Interestingly, this finding does not support the theory that prostatic neoplasia in castrated dogs is of a more aggressive nature or that castration favors tumor progression [6,30,32].

## 7. Prostate Cancer in the Human

Prostate cancer in the human (HPC) and its counterpart in the dog, although similar in many aspects, show important differences that may explain the improvement in outcome in the human and the continuing difficulty to obtain improvement in veterinary medicine [27,64]. In 1995, prostatic cancer in the human man was reported to be the most frequently non-cutaneous tumor diagnosed in the United States of America, accounting for >400,000 deaths each year [65], whereas in 2005, a rate of diagnosis of 232,090 and a number of 30,350 deaths was reported [15]. Worldwide the estimate of new diagnosis of prostatic cancer in the human accounts for 903,500 males, with a difference between more and less developed countries [66]. This difference, as well as the improvement in mortality rate, may be attributed to differences in the availability of diagnostics and treatment services [66]. The rapid rise of the number of diagnosis in the 1990s, as well as the combination of a high number of diagnosis and a low number of mortality in 2008, may be mainly attributed to the increased availability of screening tests such as the prostate-specific antigen (PSA), which permit early detection of prostatic cancer in the human [66]. One of the similarities between human and canine prostatic cancer is the age of diagnosis. The use of an algorithm that changes the chronological age of dogs into physiological age permitted the comparison between age at diagnosis of humans and dogs [67]. The mean and median of physiological age at diagnosis for CPN was 67 years and 73 years, respectively, whereas humans were diagnosed with a mean age of 70 years [67]. Further findings of high-grade prostatic neoplasia in the dog as well as the tendency to develop bone metastasis gave reason to believe that the dog may be a valuable animal model for human prostate cancer [16], especially for the late, androgen-independent stage with metastasis in lymph nodes, lung, and bone [68]. Although these similarities are important, one of the main differences between CPN and HPC is the initial hormone responsiveness of HPC [64,69]. This sensibility on testicular hormones gives the possibility of hormone ablation therapy. Instead, CPN was reported to be not influenced by hormone ablation and the absence of influence of testicular hormones may be a possible explanation for the lack of protective effect of castration towards its development [29]. Further, the high number of post-mortem diagnosis of HPC (in >40% of necropsied men) may be considered an indication for a high number of latent or slow-growing prostatic tumors that have not been reported for CPN [6,27]. 

## 8. Discussion

### 8.1. Etiopathology

The etiopathology of the canine prostatic neoplasia remains until the present day unclear. Immunohistochemical methods were used to either distinguish certain types of CPN [35] or to provide insight on possible initiating or modulating factors [53,62,70]. The work presented by Shidaifat and co-authors [70] on the expression of vascular endothelial growth factor (VEGF) and transforming growth factor-β (TGF-β) in prostates of castrated animals are in contrast with the assumption of different authors, that castration of the male dog may have an impact on the development of prostatic neoplasia. A decade after this conclusion was made, Fonseca et al. [56] hypothesized that p63-positive tumors may be a distinct entity rather than a subtype of prostatic carcinoma. This finding may be used as motivation to investigate in the future, if the tumors of castrated males are more likely to be p63-positive and may, therefore, follow a different etiopathological pathway than the neoplasia of intact males.

### 8.2. Morphology of Canine Prostatic Neoplasia

Studies published in the early 21st century started to provide information on morphological subtypes of CPN. A confounding factor in the denomination of prostatic neoplasia is the fact that studies, such as Bryan et al. [15] included the transitional cell carcinoma (TCC) in the group of prostatic neoplasia. Studies that do not provide information on the morphological subtypes of CPN also did not state whether they included or excluded the TCC of the bladder and/or the prostatic urethra [1,6,24,59]. Inclusion of TCC into the group of CPN may result in an incorrectly calculated or reported incidence of prostatic neoplasia in the dog. MacLachan et al. [71] report PACA and TCC as the main neoplasia found in the canine prostate. Due to its different cellular origin, TCC should be considered a neoplasia of non-prostatic origin which has to be excluded from statistical evaluations of incidence, outcome and MST of prostatic neoplasia in the dog. Knowledge about the morphological subtypes is of importance due to the suspected difference in biological behavior, such as metastatic behavior [32,38], and the difference in frequency in the groups of intact and castrated male dogs [30,32,48,55]. Leav and Ling [27] provided very early in prostatic research, a classification of the morphological subtypes, which remains valid until the present day. The most common morphological subtype in both intact and castrated animals is the intra-alveolar proliferative adenocarcinoma (Type A1) [33]. Gobello et al. [48] instead report that castrated males are more often affected by poorly differentiated prostatic neoplasia. Cornell et al. [32] and Palmieri et al. [38] report a trend towards an increased overall metastatic frequency of tumors of mixed morphology. It is due to these results that we consider the information on the morphological subtype vital for further research.

### 8.3. Signalment and Clinical Signs

For both owners and veterinarians, it is important to know the groups at risk for CPN development and their clinical presentation. First of all, age seems a factor of prime importance in the development and diagnosis of prostatic neoplasia in the dog, just as it is in men. The mean age at diagnosis ranges in the various studies between 8.5 years and 11.2 years [1,6,16,23,24,27,28,29,39] (Table 1). In most studies, dogs affected by CPN are reported to be either six years of age or older [1,6,15,23,24,27,30,32]. Polisca et al. [39] suggest further to start with a geriatric screening of the prostate in male dogs from 6–7 years on. Additionally, BPH is a prostatic disorder mainly found in elderly dogs and is found with increasing frequency with increasing age. Therefore, a correlation between the changes during aging and the development of both BPH and prostatic neoplasia may be hypothesized [48]. BPH and prostatic neoplasia share therefore similarities not only in the age distribution but may be present concomitantly in intact male dogs [49]. Nevertheless, the presence of BPH is not a predisposing factor for the development of prostatic neoplasia [42]. Although few studies provide information on the mean or median body weight of dogs diagnosed with prostatic neoplasia, the majority of researchers report a population of mainly medium- to large size dogs [1,21,22,32,40]. Breed predispositions are widely negated, yet dogs of Shetland Sheepdog, Scottish Terrier, Bouvier des Flandres, Dobermann Pinscher, and mixed breeds are mentioned more frequently than other breeds [6,15,23,24]. Due to the overrepresentation of medium to large size dogs in many studies, we suggest consideration of dogs of medium to large size over six years of age as at increased risk for CPN regardless of their sexual status. In dogs, clinical signs caused by prostatic neoplasia have been reported as well for other prostatic disorders, and may therefore not be considered pathognomonic [24,41]. However, both in CPN as well as in other prostatic disorders, clinical signs may easily be explained by the topographical location of the gland. Neoplasia of the prostatic gland has been reported to be locally invasive and capable of compromising surrounding organs with increasing size [24,28,29]. Urinary tract signs, although possibly present in other prostatic disorders, are often reported as the prime clinical signs in dogs affected by CPN [1,23,24,32,38]. Locomotor and systemic signs in dogs with CPN may be considered in a high percentage of cases as caused by metastatic disease or progressed local infiltration of the prostatic neoplasia [32,41]. Alterations found during digital rectal palpation may indicate the presence of a prostatic disorder, yet none of these alterations may be considered pathognomonic for CPN [1]. An easily palpable prostatic gland in a dog that was castrated early in his life may already be considered a pathological finding [49]. Although digital rectal palpation is an inexpensive clinical tool, it may provide information indicating the prostate gland as a source of the clinical signs. We, therefore, advise performing digital rectal palpation during every general examination of a male dog, regardless of its sexual status. Ultrasound examinations and radiographic studies are useful tools to evaluate the structure, size and presence or absence of prostatic or paraprostatic cysts [23,30,41]. Calcification of the prostate is frequently found in diagnostic imaging of CPN [30,41]. Although the finding of calcifications is supportive of a suspected diagnosis of prostatic neoplasia, they may be present also in other prostatic disorders [72]. The only reliable tool to confirm the suspected diagnosis of CPN remains the histological or cytological evaluation of prostatic specimens obtained by various methods [1,27]. We regard fine-needle aspiration biopsies as not advisable in routine examinations of dogs at risk of CPN, yet necessary in cases of a substantial suspect of the presence of CPN. Therefore, we advise routine general clinical examination of at-risk dogs in the absence of clinical signs, including rectal palpation and diagnostic imaging [39]. 

### 8.4. Diagnosis

Although advances in the understanding of prostatic neoplasia have been made, the search for a diagnostic marker for CPN, similar to the PSA serum concentration in men, continues [23,30,57]. The availability of such an early marker may improve overall outcome, considering that today 60–80% of dogs are already affected by the gross metastatic disease at the time of diagnosis [29,32]. Until an early marker is discovered and validated, we consider screening ultrasound examinations in at-risk dogs recommendable and in case of suspected CPN, a cytological or histopathological examination should be performed. To the present day, cytological and histopathological examinations of prostatic specimens remain the only methods to confirm a suspected CPN [1,27,41]. In human medicine, the Gleason score is a widely used and recognized method to predict the prognosis of prostatic cancer [16,73,74]. Palmieri et al. [43] applied this method in cases of CPN, suggesting that the Gleason score and grading system may be useful in prognostic prediction also in the dog. Nevertheless, the presence or absence of metastasis remains a very important factor in prognostic evaluations and ultimately influences also the choice of treatment [22,40,43]. In the dog, the organs most likely affected by a metastatic disease are the regional lymph nodes and the lung [1,22,27,30,32,34,43]. A radiographic study or if possible, a computed tomography (CT) scan, should be included in the staging of CPN, and may potentially be performed also before cytological or histological results are available [22,32,40]. Considering the frequency of urinary tract signs, we consider it useful to include a urin analysis in the clinical protocol to rule out concomitant urinary tract infections or other problems related to the urinary bladder or the lower urinary tract [30]. Considering the improvement in mortality rate in human prostate cancer following the initiation of screening examinations using an early marker, we consider the search for an early marker of utmost importance towards the improvement of prognosis in CPN. 

### 8.5. Treatment and Prognosis

Treatment of prostatic neoplasia consisted initially in castration, estrogen therapy and therapy with antibiotics and NSAIDs [22,30,41]. Although castration may have a beneficial effect on clinical signs due to the reduction in glands size, ultimately it does not impact the survival time of the animal. The reduction of prostatic size and, subsequently, the improvement of signs may be mainly due to the concomitant presence of BPH in intact dogs with prostatic neoplasia [49]. Total prostatectomy and subtotal prostatectomy showed a beneficial impact on the survival time of the animals, yet mainly, if not only, in cases without metastasis [40,59]. Chemotherapy became more frequently used in veterinary medicine over the last decades for various neoplastic disorders. However, the intensity of the treatment plan, its economic cost and the not yet widely acknowledged usefulness in terms of curative outcome, make it a rare treatment choice [10,22]. Ravicini et al. [22] used chemotherapy in combination with NSAIDs and supportive therapy in their CPN cases. Just as in Bennett et al. [40] and L’Eplattenier et al. [59], the most important factor of impact on the MST was the presence or absence of metastasis at the time of diagnosis. Although the MST was prolonged up to over seven months in dogs without metastatic disorder at the time of diagnosis, the patients ultimately died or were euthanatized due to prostatic neoplasia and its complications. The high percentage of metastatic disease at the time of diagnosis highly influences the decision of the owner to opt for euthanasia at the time of diagnosis or shortly after. In the study of Obradovich et al. [29], 81% of the owners refused treatment. Clinical signs such as pain and discomfort obviously should be treated and euthanasia should be performed if the dog’s suffering exceeds his quality of life, yet to the present day, no data is available on how long dogs may survive if adjuvant therapy is the only treatment they receive.

### 8.6. Castration

Castration is a routinely performed surgery in the dog, mainly but not exclusively to avoid unwanted offspring. Removal of the testicles subsequently results in the removal of testosterone and its active metabolite DHT from the general circulation. The removal of these hormones results in a decrease in the size of the prostatic gland and a decrease in sexually motivated behavior and infertility [41,75]. Hormonal implants have a similar yet reversible effect on the canine organism [76], yet until today, no studies have been designed to evaluate a possible increased risk for tumor development in implanted, and therefore chemically and reversibly castrated animals. Considering that more than 95% of dogs over the age of nine years are or will be affected by BPH, castration is also used for treatment and/or prevention of this disorder [48]. Surgical castration causes a 70% reduction in the dimension of the gland. Although this process begins already 7–14 days after castration, complete involution of the gland may be expected by four months [41,77]. Dubé et al. [78] report that, although the reduction in size in dogs affected by CPN was less than in unaffected dogs, it was nevertheless present. This decrease in size may be attributed mainly to the reduction of epithelial cells, followed by basal cell proliferation [79]. Considering that prostatic acini are the androgen-responsive part of the prostatic gland, ductal and urothelial tissue is not affected by castration [34]. Knowledge of the reduction of prostatic size and volume after castration makes reports of a high risk of prostatic neoplasia in castrated dogs a surprising finding. Although different authors provide information on the incidence of castrated dogs in their studied population, no unanimity can be found when considering the impact castration might have on the development of prostatic neoplasia. L’Eplattenier et al. [59] report that castration not only has no effect on the progression of the disease but it does not even prevent the occurrence of prostatic neoplasia. The same may be said about Obradovich et al. [29] and Bell et al. [30] who include castrated animals in their population, yet conclude that an association between castration and prostatic neoplasia cannot be determined. Cornell et al. [32] are the first to hypothesize a potential effect of castration on the progression of the neoplasia from androgen-dependent to an androgen-independent state. They consider neither castration as preventive for the development of prostatic neoplasia nor life-time testis exposure as essential for the development, yet suggest a decreased risk in dogs castrated before the age of six months. In 2002, Teske and co-authors [6] follow and support the hypothesis on the impact of castration on tumor progression in their study conclusions. Bryan et al. [15] find the clearest words by describing a highly significant association between castration and the development of prostatic neoplasia. Nevertheless, they acknowledge the positive impact of castration in terms of lifetime. Considering the lack of information on median survival time, metastatic behavior and other outcome-related factors within the study of Teske et al. [6] and the fact that MST did not differ, or differed in a positive manner between castrated and intact male dogs in other studies, it remains difficult to understand on the basis of which data a favorable influence of castration on tumor progression may have been suggested [6,32]. After a thorough evaluation of the data presented in the various publications, the only conclusion that can be made is that there is no protective effect of castration for the development of prostatic neoplasia. Several aspects should be considered in the evaluations of these studies, such as the differences in study design, the differences in the studied population and most importantly the different definitions on when a dog is to be considered castrated. Although some authors provide information on the CDI within their studied population, either in the form of a mean, median or a range, the minimal CDI necessary to consider an animal castrated at the time of diagnosis is a value mostly neglected. In the few studies in which this minimal CDI is described, the value differs greatly [6,29]. A minimal distance between castration and diagnosis of CPN of 100 days seems to us far too little to ensure the absence of neoplastic cells at the time of castration. We consider the classification used by Obradovich et al. [29] as appropriate, easy to understand and very informative. We cannot underline enough the importance of such information and it seems inevitable that a minimal CDI should always be reported in future studies on the subject in order to reasonably rule out the possibility that the neoplasia was already present at the time of castration. Castration was and is by most still considered a very important tool in the prevention and long-term treatment of the most common prostatic disorder, BPH [21]. Comparing the incidence of BPH and prostatic neoplasia, it is obvious that clinicians will be more often confronted with BPH and its treatment than with prostatic neoplasia. Shidaifat and co-authors [70] conclude that their results do not agree with the hypothesis that castration plays a role in prostatic neoplasia development. This finding may be considered supported by L’Eplattenier et al. [59], who describe that castration has no effect on tumor progression although gonadectomy has no protective effect against the disorder. On the contrary Heuter et al. [80] acknowledge an increased risk for castrated dogs, yet consider the risk so low that it should be regarded as clinically irrelevant. Obradovich et al. [29] conclude that castration had no influence on the development of prostatic neoplasia in their studied population and Krawiec and Heflin [24] further pointed out that most prostatic disorders, e.g., BPH, may be prevented by surgical castration; a conclusion supported also by Sorenmo et al. [34]. It is mainly Teske et al. [6] and Bryan et al. [15] who consider the impact of castration on prostatic neoplasia important, suggesting castration a possible promoter from an androgen-dependent to an androgen-independent state of a tumor. Bryan et al. [15] describe the association between castration and prostatic neoplasia development as highly significant, yet point out that the higher median age at diagnosis shows a benefit of gonadectomy in terms of lifetime. Although intact male dogs may be affected by numerous prostatic disorders (e.g., prostatitis, BPH, abscess etc.), castrated males are rarely affected by prostatic pathologies. In the rare case of a castrated male presenting with a prostatic disorder, it is significantly more often a CPN rather than any other of the prostatic disorders [39]. Considering all previous reports, the assessment of Polisca et al. [39] can hardly be negated as in fact, prostatic neoplasia is one of the few prostatic disorders that may be found in castrated males. Although these results show that castration has no protective effect against the development of CPN, it may nevertheless be considered proven that there is a beneficial effect of castration on the prevention of other prostatic disorders. Until today, there is neither proof of castration having an impact on the development and/or progression of prostatic neoplasia in a positive or negative fashion, neither is there any scientific evidence that castrated dogs are affected by a more or less aggressive type of prostatic neoplasia. The impact of a gonadectomy on the development of prostatic neoplasia is on the basis of current knowledge that is difficult to assess. We consider it most interesting that, although odds ratios and increased risks may have been statistically proven in different studies [6,29,30,32], only a few authors conclude that there is, in fact, an association between castration and CPN development. Nevertheless, the age of the dog may be taken into consideration when programming an elective gonadectomy. In most studies, dogs affected by CPN are either six years of age or older [1,6,15,23,24,27,30,32]. These reports give reason to believe that castration of adult male dogs under the age of six years may be performed with a very low risk of concomitant presence of CPN, whereas castration of dogs over the age of six years may be considered after a thorough evaluation of the prostate. The same may be said on immunohistochemical findings and findings of metastatic behavior. It has been reported that positivity for CK-7 was associated with a higher frequency of metastatic disorder (in particular a higher frequency of bone metastasis). Further, dogs with CK-7 positive tumors have been reported to have been castrated at a younger age. Nevertheless, these findings do not provide enough evidence to support the statement that dogs castrated at a younger age suffer from a more aggressive type of CPN, not even for the authors of the study themselves [34]. We consider this caution in drawing conclusions of major importance regarding the possible influence of castration in the context of CPN development. Sadly, this caution is often missing when results are presented and cited in experimental studies as well as in reviews. Unfortunately, it is not uncommon to find citations in review papers (cited in this review) which are not completely in line with what was stated in the cited reference. Furthermore, the presentation of odds ratio and the increased risk is difficult to be interpreted in the context of a scientific review without information on the study design, inclusion criteria, and conclusions drawn by the authors. Conclusions that may be drawn on the basis of results taken out of context may have an important effect on different stakeholders, e.g., pet owners or veterinary practitioners. Gonadectomy in both male and female dogs has been further linked to other possible side-effects, such as incontinence [81] and obesity [82]. Furthermore, studies have reported an increased risk for the development of osteosarcoma [5], mastocytoma [8], lymphoma [7], hemangiosarcoma [4], and different orthopedic conditions [83]. Similar to the reports on an increased risk of CPN development in the castrated male dog, these studies also have to face important limitations. Some authors limit their evaluation on specific breeds [83,84] which may be a confounding factor for the results and conclusions. Yet the limitation we consider as the most important one is the lack of information on the castration-diagnosis-interval. The majority of studies classifies the population as either spayed and intact [4,7,8,85] or early-spayed, late-spayed and intact [83,84]. The CDI, as has been described above for CPN may be considered to be of the same importance for other neoplastic disorders to suspect or hypothesize a causal relationship between gonadectomy in the dog and cancer development. 

## 9. Conclusions

Within the last two decades, castration was defined as a risk factor not only for prostatic neoplasia but for other neoplastic disorders in both males and females, causing an international discussion if elective gonadectomy in dogs is still a tool that should be used routinely. However, there is currently not enough evidence supporting an increased risk of developing prostatic neoplasia after castration to take a decision against elective gonadectomy in male dogs. More research is needed on this topic and, most importantly, scientific papers on this topic should always provide detailed information on (a) whether or not dogs were castrated, (b) when castration was performed during life, (c) the size of the reference population, (d) how long before the diagnosis the dog was castrated in order to be included in the castrated group. In healthy dogs, gonadectomy should be considered a reliable and valuable preventive treatment for BPH and other non-malignant prostatic disorders. On the basis of the data provided by the currently available scientific literature, elective gonadectomy of adult male dogs of under six years of age cannot be excluded from the daily veterinary practice due to concern of causing prostatic neoplasia until clear and strong evidence is available.

## Figures and Tables

**Table 1:** Overview of the studied populations and the signalment in chronological order of publication.

Studies	N_total_	N_PD_	N_CPN_	Age (y)	BW (kg)	Prevalence	I/C_t_/C/U	CDI (Years)
[25]	>500		3			0.6		
[19]	7248		8			0.11		
[26]			6					
[27]			20	10.1 ^A^			16/3/1/0	
[28]		140	22	9.3 ^A^			0/0/1/21	
[1]	~4500	430	15	9 ^A^	27.8 ^A^		15/0/0/0	
[23]	1483/13,633		14	8.5 ^A^		0.94/0.05	7/0/7/0	0.75-5 ^E^
[29]			43	9.8 ^A^			14/10/19/0	GI: 5.5 ^C^; 7 ^D^ GII: 9.6 ^C^;6.5 ^D^
[30]			31	10 ^B^			21/0/10/0	6.4 ^D^
[24]	7069	177	13	10 ^A^		0.18	5/1/7/0	2–8 ^E^
[31]			15				4/0/11/0	
[16]	199		25	9.4 ^A^		12.6	4/0/11/10	
[32]			76	10 ^B^	20.5 ^B^		28/12/36/0	7 ^D^
[33]			19				9/0/10/0	
[6]	15,363	431	56	9.9 ^A^		0.3	30/0/26/0	1–10 ^E^
[34]			70	10 ^B^			21/0/49/0	
[35]			17				5/0/12/0	
[36]	8179		2			0.02		
[37]		25	3				1/0/2/0	
[15]			1384					
[38]		111	50					
[17]	26	18	5			19.23		
[39]	72,300	418	11	11.2 ^A^		0.015	7/0/4/0	
[18]	61	51	29			47.5		
[40]			10	9.3 ^B^	25 ^B^		0/0/10/0	
[22]			67	9.5 ^B^	23.3 ^B^		7/0/60/0	

N total = total number of animals in the population; NPD: number of animals with prostatic disorder; NCPN: number of animals with prostatic neoplasia; y = years; BW = body weight; A = mean; B = median; P = prevalence calculated based on total number of animals divided by number of dogs with CPN; I = intact; CT = castrated as treatment; C = castrated; U = unknown; CDI = castration-diagnosis-interval; GI = Group I (castrated >12 months of age and >3 years prior to diagnosis); GII = Group II (castrated <12 months of age and >3 years prior to diagnosis); C = mean CDI; D = median CDI; E =range of CDI.

**Table 2:** Percentage of clinical signs in dogs affected by prostatic neoplasia in the respective studied population.

Studies	GI (%)	Urinary Tract (%)	Locomotor (%)	Systemic (%)
[27]	45	65	55	70
[1]	60	73	27	33
[30]	45			35
[24]	31	61	7.7	23
[32]	30	62	36	42
[38]	22	30	16	16
[43]	7.5	50	12.5	5
[39]	82			46
[40]	24	96		32

GI = gastro-intestinal.

**Table 3:** Frequency of different histological types of canine prostatic neoplasia in the respective studied population.

Studies	Adenocarcinoma (%)	Undifferentiated Adenocarcinoma (%)	Carcinoma (%)	Mixed-Morphology (%)	Other (%)
[27]	80	20			
[32]	36			53	11
[15]	43		29		28
[55]			25	75	
[38]			62	38	
[22]	54				

**Table 4:** Frequency and site of metastasis in dogs affected by prostatic neoplasia in the respective studied population.

Studies	Iliac Ln (%)	Lung (%)	Bone (%)	Urinary Bladder (%)	Other Organs (%)	Total Frequency
[27]	75	65	35	50	5–35	15/20 dogs
[1]	87	33	20	60		13/15 dogs
[30]	33	62	15	18	11–33	13/25 dogs
[20]			24			
[32]	51	50	22		1–9	61/78 dogs
[34]	43	32	25.5			45/70 dogs
[38]	33.3	41.7			8.3–25	12/50 dogs
[43]	43	43	14			14/28 dogs
[22]	28	15	2		1	26/67 dogs

Ln = lymph node.

**Table 5:** Illustration of possible treatments of CPN.

Studies	Primary Treatment	Adjuvant Treatment	N Treated Animals	MST
[59]	Partial prostatectomy	Local interleukin-2 and systemic meloxicam	8	5–239 d (103 d)
[22]	NSAIDs; NSAIDs and chemotherapy; Chemotherapy	Tramadol, maropitant, lactulose, amantadine, mirtazapine, gabapentin	67	9–752 d (82 d)
[40]	Total prostatectomy	NSAIDs, opioids, tramadol, ketamin or acetaminophen	25 (15 TCC, 9 carcinoma, 1 cystadenocarcinoma)	TCC: 34–664 d (189 d); Adenocarcinoma: 24–1255 d (248 d)

NSAIDs = nonsteroidal anti-inflammatory drugs; N = number; TCC = transitional cell carcinoma; MST = median survival time; d = days.
